# Hyaluronan Hydrogels for Injection in Superficial Dermal Layers: An In Vitro Characterization to Compare Performance and Unravel the Scientific Basis of Their Indication

**DOI:** 10.3390/ijms22116005

**Published:** 2021-06-02

**Authors:** Annalisa La Gatta, Maria Aschettino, Antonietta Stellavato, Antonella D’Agostino, Valentina Vassallo, Emiliano Bedini, Gilberto Bellia, Chiara Schiraldi

**Affiliations:** 1 Department of Experimental Medicine, School of Medicine, University of Campania Luigi Vanvitelli, Via L. De Crecchio 7, 80138 Naples, Italy; maryaschettino75@gmail.com (M.A.); antonietta.stellavato@unicampania.it (A.S.); antonella.dagostino@unicampania.it (A.D.); valentina.vassallo@unicampania.it (V.V.); 2 Department of Chemical Sciences, University of Naples Federico II, Complesso Universitario Monte S. Angelo, Via Cintia 4, 80126 Naples, Italy; ebedini@unina.it; 3 IBSA Farmaceutici Italia, 26900 Lodi, Italy; gilberto.bellia@ibsa.it

**Keywords:** glycosaminoglycans, hyaluronan, dermal filler, hydration, rheological behavior, hyaluronidase, degradation by reactive oxygen species, human dermal fibroblasts

## Abstract

Background: Skinboosters represent the latest category of hyaluronan (HA) hydrogels released for aesthetic purposes. Different from originally developed gels, they are intended for more superficial injections, claiming a skin rejuvenation effect through hydration and possibly prompting biochemical effects in place of the conventional volumetric action. Here, three commercial skinboosters were characterized to unravel the scientific basis for such indication and to compare their performances. Methods: Gels were evaluated for water-soluble/insoluble-HA composition, rheology, hydration, cohesivity, stability and effect, in vitro, on human dermal fibroblasts towards the production of extracellular matrix components. Results: Marked differences in the insoluble-hydrogel amount and in the hydrodynamic parameters for water-soluble-HA chains were evidenced among the gels. Hydration, rigidity and cohesivity also varied over a wide range. Sensitivity to hyaluronidases and Reactive Oxygen Species was demonstrated allowing a stability ranking. Slight differences were found in gels’ ability to prompt elastin expression and in ColIV/ColI ratio. Conclusions. A wide panel of biophysical and biochemical parameters for skinboosters was provided, supporting clinicians in the conscious tuning of their use. Data revealed great variability in gels’ behavior notwithstanding the same clinical indication and unexpected similarities to the volumetric formulations. Data may be useful to improve customization of gel design toward specific uses.

## 1. Introduction

Facial injection of hyaluronan (HA)-based gels for aesthetic purposes is well-established and HA crosslinked with 1,4 butandiolediglycidylether (BDDE), suspended in physiological solution, is generally employed [1,2,3,4,5,6,7,8,9,10,11,12,13,14,15,16,17,18,19,20,21,22,23,24,25]. The use of the BDDE-HA hydrogel typically relies on its elastic behavior, high hydrophilicity and improved resistance to hyaluronidase action, compared to unmodified HA. The elastic behavior is responsible for gel capacity to maintain volume while deforming under the stress of facial movements thus assuring a filling, natural-looking effect; network hydrophilicity improves tissue hydration at the injection site while the higher (but not complete) resistance to enzymatic degradation, compared to natural-occurring HA, assures longer in vivo permanence while preserving bio-absorption.

Since the launch of the first products, the suggested clinical use for this type of hydrogels has changed. Volume restoration, based on the gel physical “filling” effect, has been considered the main indication for more than two decades [2,10,11,12,13,14,19,22]. Considering that gel-projection capacity is related to its stiffness, G’ (Storage Modulus) has long been the only parameter considered in selecting the most suitable gel for the specific clinical need. A more recent approach has been extended to the whole fluido-dynamic gel behavior as well as to its sensitivity to enzymatic and radical degradation and to other properties (e.g., hydration capacity, cohesivity) [1,5,6,7,8,14,15,16,17,18,19,20,21,22,23,24,25]. All of the latter features are known to contribute to the in vivo effect after injection and/or over time, and they are therefore generally studied to predict relative in vivo performance and to provide clinicians with valuable information to select treatments and optimize outcome [1,5,6,7,8,14,15,16,17,18,19,20,21,22,23,24,25]. Additionally, due to these studies, HA-BDDE gel design has been improving. As a consequence, hydrogels are now tuned towards more specific and differentiated uses to meet diverse clinical needs finally aiming at a full face restoration [7,8,21,26,27]. “Skinboosters” are the latest category of HA gels that entered the market. Unlike typical “volumetric” fillers, skinboosters are intended for the injection into more superficial (intradermal vs. deep dermis-periosteum) skin layers and are expected to improve skin appearance and texture rather than performing by a projection effect. Specifically, improvement in skin hydration and elasticity, and possibly a boost in extracellular matrix molecules biosynthesis is claimed after the application of such gels [28,29,30,31,32,33]. A stretching effect, referring to wrinkle distension from gel hydration, that is anyway filling the volume loss to a certain extent, is also predicted [30].

Former studies and literature report extensively analyzed volumetric gels and compared available formulations, suggesting opportunities toward design optimization, and supporting their appropriate selection and use [1,5,6,7,8,14,15,16,17,18,19,20,21,22,23,24,25]. On the contrary, no similar studies have been carried out so far to scientifically assist this recent “skinboosting” approach. The aim of the study was to assess a panel of biophysical and biochemical features for this specific type of hydrogel. For this purpose, three gels among the currently marketed crosslinked HA-based skinboosters (i.e., Restylane Vital (R_v_), Juvederm Volite (J_V_), Viscoderm Hydrobooster (H_B_)) were evaluated for their composition in water-soluble/insoluble HA, rheological behavior, hydration capacity, cohesivity, sensitivity to degradation and ability to induce, in vitro, human dermal fibroblasts towards enhanced production of extracellular matrix components. Beyond increasing our knowledge of these gels, results allow the comparison of gel performance. Further, they may shed light on the possible specific features for the HA-BDDE gels to be classified as a skinbooster

## 2. Results

### 2.1. Water-Soluble Fraction of Fillers: Quantitative Determination and Hydrodynamic Analysis

Total HA concentration in each gel is provided by the manufacturer (Table 1). The water-soluble fraction of the biopolymer was quantified here, allowing us to derive the specific composition in water-insoluble/water-soluble HA (Table 1). Water-soluble HA was found in each formulation and in a comparable amount (about 4 mg/mL; *p* > 0.05). As a result of the diverse biopolymer total concentration, the gels significantly differ for the water-insoluble HA content. Specifically, the insoluble HA fraction in H_B_ was far higher than the one found in the other gels, up to 2.6 fold higher, compared to J_V_. As for R_V_, the insoluble HA content almost doubled compared to J_V_.

The SEC-TDA analyses revealed the presence of a 525 ± 23 kDa HA in H_B_ (Table 2). Shorter HA chains, of about 260 kDa *M*_w_, were found in R_V_ and HA of ~160 kDa was found in J_V_. The intrinsic viscosity and the hydrodynamic radius values varied consistently. The *M*_w_/M_n_ values suggested for R_V_ and, especially J_V_ broader distributions compared to H_B_ one.

By comparing the Mark–Houwink–Sakurada (MHS) curves (log intrinsic viscosity vs. log molecular weight) derived for the soluble fractions to the ones obtained for linear HA samples (Figure S1), a lower intrinsic viscosity over all the molecular weight distribution, was found for the analyzed samples, thus indicating a more compact conformation, consistent with modified chains. Thus, we can assert that soluble HA fractions contain BDDE-HA molecules.

### 2.2. ^1^H-NMR Analyses

The ^1^H-NMR spectra obtained for the gels are shown in the Supplementary Material (Figure S2). The BDDE/HA (disaccharide unit) molar ratio was quantified by integrating the signal at δ 1.6 ppm, due to the aliphatic (CH_2_)_2_ moiety of the BDDE molecule, with respect to the HA N-acetyl signal at δ 1.9 ppm (ref). The BDDE/HA ratio (mol %) was 1.1, 7.0 and 9.5 for R_V_, J_V_ and H_B_ respectively.

### 2.3. Hydration Capacity

**Figure 1:**
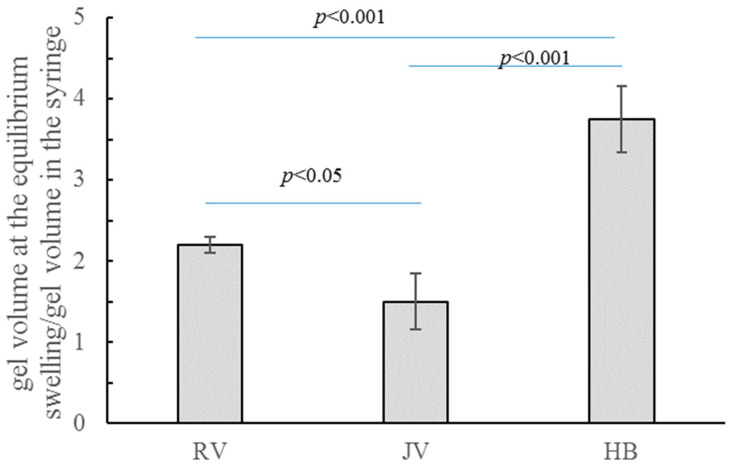
Gels’ hydration ability. Volumetric expansion (gel volume at equilibrium/gel volume in the syringe) occurring when the formulations are equilibrated in PBS; data indicate the final volume reached by 1 mL of the formulations when allowed to hydrate in PBS.

Water absorption was measured to compare the gel hydration capacity. Data reported in Figure 1 demonstrated that gels were able to hydrate and, therefore, to expand, when incubated in a physiological medium. This indicated that the commercialized formulations are not at the equilibrium swelling. Specifically, as reported in Figure 1, when allowed to equilibrate in Dulbecco’s Phosphate Buffered Saline (PBS), R_V_ almost doubled its volume and H_B_ more than tripled it. J_V_ showed the lowest hydration extent (*p* < 0.05 vs. R_V_ and *p* < 0.001 vs. H_B_).

### 2.4. Rheological Parameters

**Figure 2:**
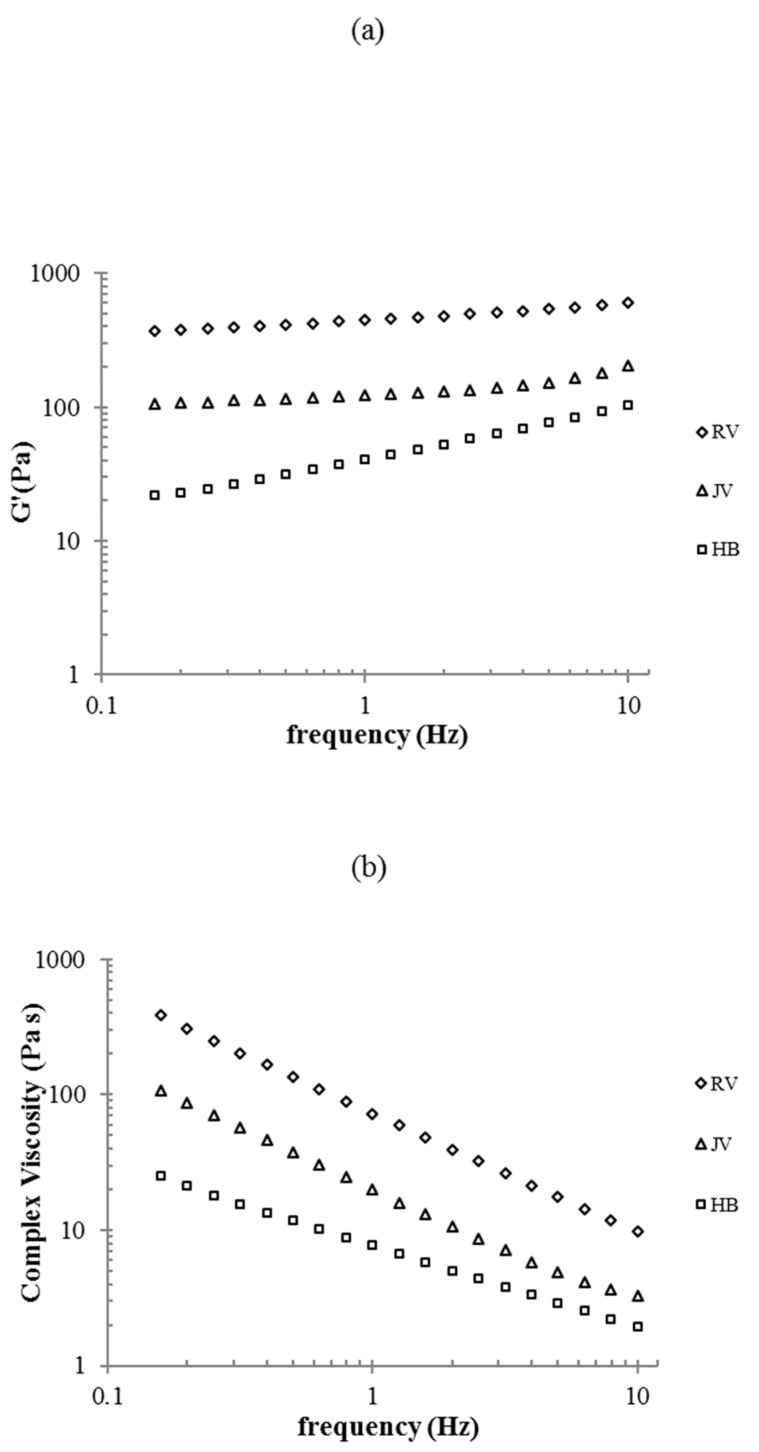
Rheological characterization. G’ values (**a**) and complex viscosity (**b**) as a function of the frequency. Measurements were performed at 37 °C and 0.1% strain.

The rheological characterization confirmed, as expected, an elastic behavior for all the gels, with tan delta values in the range 0.2–0.6. Further, results indicated that the gels were not comparable for rigidity and, surprisingly, G’ values varied over a wide range (Figure 2a). R_V_’s stiffness was far higher, while H_B_ behaved as the less rigid gel. Specifically, G’ values at 0.7 Hz frequency were about 40 Pa for H_B_, about 120 Pa for J_V_ and as high as 430 Pa for R_V_. The complex viscosity data confirmed the typical profile of this type of hydrogels with values constantly decreasing with frequency. R_V_ behaved as the most viscous gel while H_B_ proved the least viscous one (Figure 2b).

### 2.5. Degradation Studies

**Figure 3:**
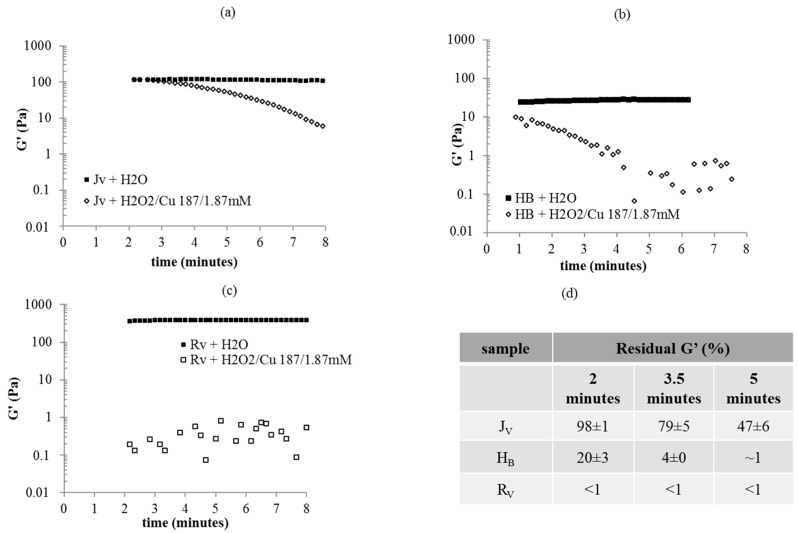
Degradation in the presence of ROS. G’ values during incubation with H_2_O_2_/Cu^2+^ 375/3.75 mM, compared to a control (gel diluted to the same extent with water) for J_v_ (**a**), H_B_ (**b**) and R_v_ (**c**). Measurements were performed at 37 °C, 1.59 Hz frequency and 0.1% strain. (**d**) Residual G’ (% in respect to G’ for the control) at 2, 3.5 and 5 min of incubation with ROS.

**Figure 4:**
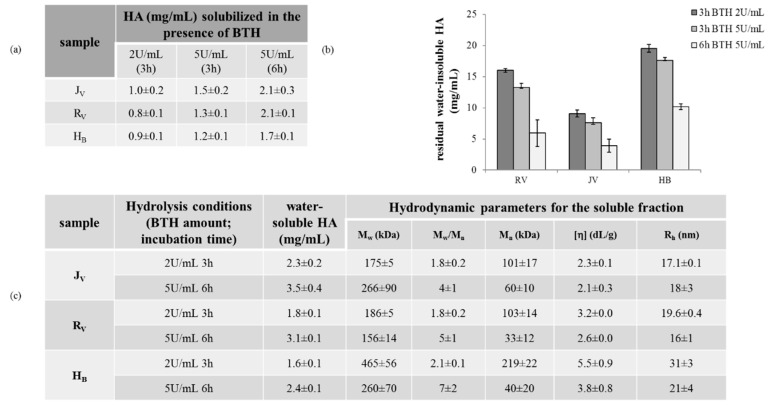
Enzymatic degradation. (**a**) Amount of HA (mg/mL) solubilized due to the BTH action after 3 h incubation with BTH 2 U/mL and after 3 and 6 h incubation with BTH 5 U/mL. (**b**) Residual water-insoluble HA (mg/mL) under the same hydrolysis conditions as in (**a**). (**c**) SEC-TDA data for the HA water-soluble fraction after 3 h incubation with 2 U/mL and 6 h incubation with BTH 5 U/mL.

The degradation studies revealed that all the gels were sensitive to both Reactive Oxygen Species (ROS) and Bovine Testicular Hyaluronidase (BTH) action (Figure 3 and Figure 4). Gel sensitivity to ROS was demonstrated by the rheological behavior in the presence of a ROS generating system (Figure 3), compared to a control. Specifically, G’ values were recorded during filler incubation with the H_2_O_2_/Cu^2+^ system and after diluting the gel, to the same extent, with water (control) (Figure 3a–c). Dilution with water reflected in a 1.1–1.4 fold decrease in G’ values, compared to the data in Figure 2 and, gel rigidity remained constant over time (Figure 3a–c). In the presence of ROS, a marked drop in G’ was observed indicating depolymerization (Figure 3a–c). The gels showed diverse degradation profiles. Specifically, R_V_’s stiffness rapidly decreased from about 400 Pa to values lower than 1 Pa, close to the minimum measurable values, thus causing a scattered signal (Figure 3c). A more gradual reduction in G’ was observed for J_V_ and for H_B_ with the latter degrading faster (Figure 3a,b). In particular, as indicated in Figure 3d, at 2 min of incubation with the ROS generating system, G’ loss (% vs. ctr) was already higher than 99% for R_v_. At the same incubation time, J_V_ still preserved 98 ± 1% of its stiffness while, for H_B_, around 20% residual G’ was recorded, compared to control. At 5 min of incubation, J_V_ still retained 47 ± 6% G’; about 93% G’ loss could be measured only at the longer time tested (8 min).

When the gels were incubated in the presence of BTH, a certain extent of HA solubilization was registered for each sample, indicating sensitivity to enzymatic hydrolysis (Figure 4a). In particular, around 1 mg/mL HA solubilized in 3 h incubation with BTH (2 U/mL) while the amount rose to 1.2–1.4 mg/mL when higher enzyme concentration was used (5 U/mL BTH) for the same time interval. Under the most drastic hydrolysis conditions tested, around 1.7–2.1 mg/mL of water-soluble fraction increase was recorded. No significant differences in the solubilization extent (*p* > 0.1) could be detected among the gels under the applied conditions, only very slightly lower solubilization of H_B_ was recorded under the most drastic conditions. However, based on these solubilization rates and on the initial gels’ composition (Table 1). H_B_ showed the highest amount of residual water-insoluble HA during incubation with BTH, regardless of the enzyme concentration and incubation time (Figure 4b).

The hydrodynamic parameters for the soluble fractions obtained after incubation with BTH are reported in Figure 4c. After 3 h incubation with 2 U/mL BTH, longer soluble chains were found for H_B_ while under the most drastic hydrolysis conditions, slightly lower *M*_w_ values were found for R_v_. With the increase in BTH concentration and incubation time, an increase of the HA amount in solution and of polydispersity and a decrease of the M_n_ values were recorded.

### 2.6. Cohesivity

**Figure 5:**
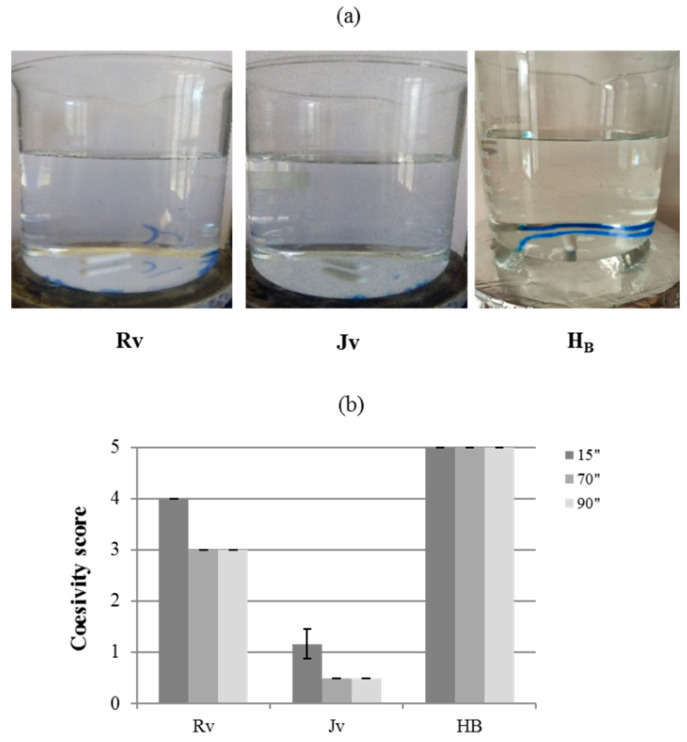
Gel cohesivity. (**a**) Images of the gels at 15 s after starting the test. (**b**) Cohesivity score assigned to the gels at diverse time intervals according to the Gavard–Sundaram Cohesivity Scale [20].

The cohesivity test revealed great differences in gels’ behavior. H_B_ completely retained its structure in the whole interval time of observation, therefore it was assigned with the highest cohesivity score (“fully cohesive”) in the Gavard–Sundaram Cohesivity Scale (Figure 5) proving the most cohesive gel. On the contrary, J_V_ progressively completely loses definition behaving as a “fully dispersed” gel (cohesivity score less than 1). An intermediate cohesive behavior was found for R_v_ that behaved as a “mostly cohesive” (cohesivity score 4) at the early time of observation then showing an increasing extent of fragmentation (cohesivity score 3, “partially cohesive” gel, at 70 and 90 s of observation).

### 2.7. Biological Studies

**Figure 6:**
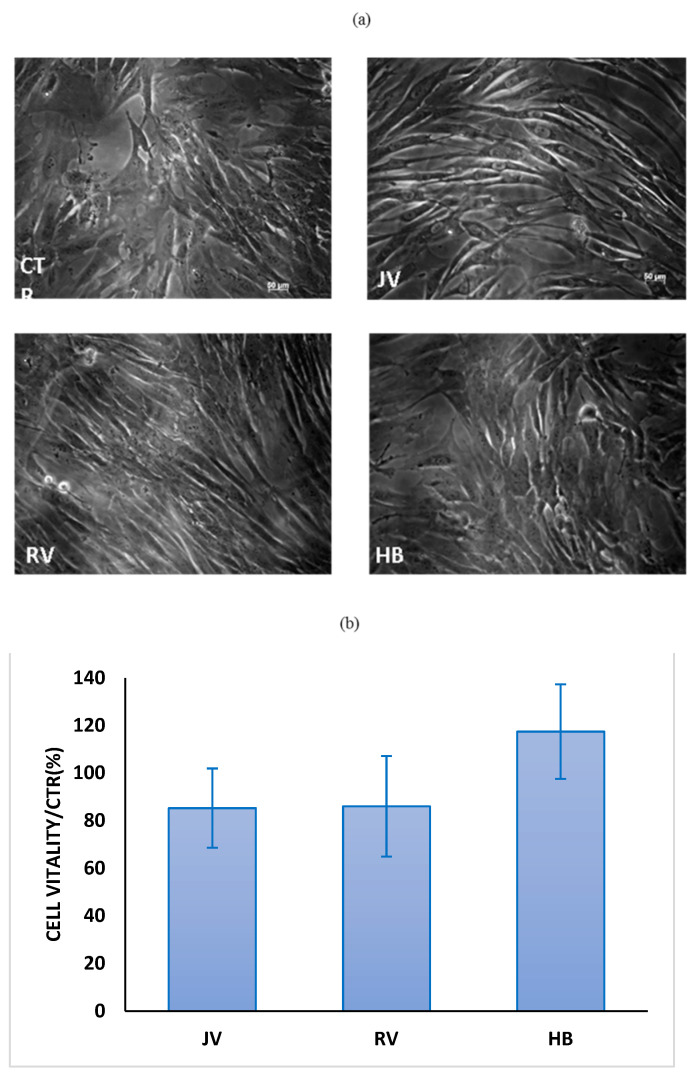
(**a**) Morphological observation at MO of Human Dermal Fibroblasts (HDF) in the control and in presence of filler, after 24 h treatment. Scale bar 50 µm; (**b**) Cell vitality with respect to the untreated cells in presence of J_V_, R_V_ and H_B_ respectively.

As shown in Figure 6, the gel treatments on fibroblasts showed the absence of any toxicity. Images of cells after 24 h incubation with the gels and of untreated cells are shown in Figure 6a. It was evident that fibroblasts in presence of filler, present a very similar morphology to the untreated cells and also the cell density in each well was comparable. Quantitative results on metabolic activity, reported in Figure 6b, confirmed the total absence of cytotoxicity for all the gels.

**Figure 7:**
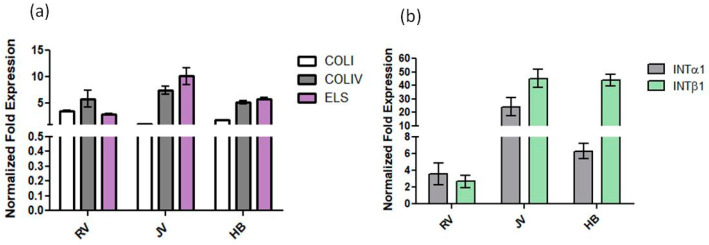
Gene expression analyses: the results are expressed as fold change of HA treated cells respect to untreated cells (CTR) for COLI, COLIV and ELS (**a**) in human dermal fibroblasts. In addition, INTα1 and β1 (**b**) were accomplished. Data showed as the averages ± SD.

To elucidate the effect on matrix synthesis due to HA treatments on human dermal fibroblasts, Type I collagen (COLI), type IV collagen (COLIV) and elastin (ELS) gene expression was quantified by qRT-PCR (Figure 7). Overall, the three biomarkers investigated were upregulated, specifically for H_B_ and R_V_ with respect to untreated cells. COLI was about 2.5-fold and 1.5-fold expression vs CTR in H_B_ and R_V_ treated cells. On the contrary, it was down-regulated by JV. COLIV was upregulated by about 3-fold, 4.5-fold and 5-fold expression over the control following H_B_, J_V_ and R_V_ treatments, respectively (Figure 7). Finally, ELS increased its expression by about 6-fold, 5.8-fold and 3.8-fold expression in fibroblasts treated with H_B_, J_V_ and R_V_ respectively. Regarding integrins, mRNA expression showed that all samples increased integrins expression (INTα1 and β1). Specifically, R_V_ upregulated INTα1 by about 4-fold and INTβ1 by 3-fold expression. Instead, INTα1 increased by about 30-fold and 7-fold vs. CTR in presence of J_V_ and H_B_, respectively. While, INTβ1 mRNA resulted in about a 50-fold increase vs. CTR for both J_V_ and H_B_, samples.

**Figure 8:**
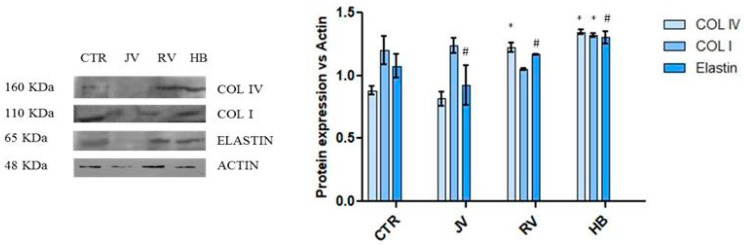
Western blotting for expression of collagen I, collagen IV and Elastin normalized to actin in the densitometry reported as average and SD. * stands for significant (*p* < 0.05) variations respect to CTR, # stands for *p* < 0.05 for elastin expression in HB treatment vs. R_V_ and J_V_ ones.

At 48 h of incubation, HDF were lysed to accomplish Western blotting analyses on specific biomarkers. Results are reported in Figure 8. The graph shows a slight increase in elastin expression in response to R_V_ and H_B_ treatments (respectively 1.09 and 1.31-fold vs. CTR), the latter proved significant vs. control and J_V_ treatment. R_V_, being less effective than H_B_ proved superior with respect to elastin biosynthesis in J_V_ treatment. This latter showed Col I production to a similar extent with respect to the control, while H_B_ slightly upregulated its expression (1.20-fold vs. CTR). Finally, the cells treated with R_V_ and H_B_ presented a Col IV higher expression in comparison to control and also J_V_ treated cells. Specifically, R_V_ increased Col IV protein level by about 1.38-fold vs. CTR and H_B_ improved it by about 1.52-fold instead, J_V_ seemed to be not effective in the modulation of this biomarker.

## 3. Discussion

Since the first application of hyaluronic acid (HA) injectable implants, the purpose for these treatments has been widened and patients and clinicians now share more consciousness. The final aim of the clinician to satisfy patient expectations without radically changing their face but improving their aspect is obtained by searching for the right product, and the right injection technique within a proposed line of formulations. It has to be considered that the biological diversity of patients and everyday life habits are the major responsible for treatment effectiveness: age, kind of skin, weight loss (or fat compartment), smoking attitude, UV irradiation, have to be considered. However, it has been established that a more precise biophysical description of the HA-based formulations may help in the right product choice according to patient needs. In this respect, scientific evidence has been improving not only to address rheological behavior or chemical aspects (e.g., HA modification degree, molecular weight, etc.) but also biochemical and biological features of the product itself. To this aim, three hyaluronan-based hydrogels were characterized in this research study to bridge the biophysical and biochemical properties to their potential performance as skinboosters and to compare them.

With regard to the gels’ composition, according to the labels, the three skinboosters greatly differ in total HA concentration [31,32,33]. The amount of total HA even doubles from one gel to another. As found for other similar formulations intended for skin rejuvenation, a certain amount of water-soluble HA was present (Table 1 and Table 2) [7,8]. In particular, data indicated comparable concentration in soluble HA (about 4 mg/mL) among the skinboosters but diverse hydrodynamic parameters. Specifically, R_V_ and J_V_ soluble HA chains were more similar in length (160–270 kDa *M*_w_) while, the soluble HA fraction in H_B_ presented far higher molecular weight (525 kDa *M*_w_), therefore potential diverse biochemical effect could be expected for this gel [34]. For all the samples, the analyses of the MHS curves suggested water-soluble HA chains conformation other than linear thus indicating a certain extent of chemical modification. This is in agreement with previous studies on similar products [15]. Rationally, the water-soluble HA fraction may derive from the crosslinking process leaving HA chains that, even if crosslinked/modified, are still soluble in an aqueous medium due to low(er) molecular weight.

Gel composition studies also highlighted, among the skinboosters, a far higher amount of water-insoluble HA (hydrogel fraction) for H_B_ thus suggesting potential different biophysical behavior for this gel. The hydrogel fraction in H_B_ was far higher even compared to volumetric gels while, in general, similar or lower water-soluble HA concentrations were found in the skinboosters [7]. Collected data indicated an unexpected similarity (in total HA concentration, composition and soluble HA chains’ hydrodynamic parameters) between the “volumetric” and the “skinbooster” Restylane formulations [7]. Even if less concentrated in total HA, insoluble/soluble-HA composition Juvederm skinbooster resulted also similar to the corresponding volumetric gel with comparable hydrodynamic parameters, as well [7].

Data on gel-water uptake support the claimed in vivo tissue hydration effect. The gels were able to absorb water even more than tripling their weight/volume (H_B_). However, it is worth underlining that J_V_ increased its volume only by about 30% that was unexpected for the pursued application as a skinbooster. This result is peculiar if compared to the water up-take values generally reported for other dermal fillers with volumetric indication (up to 280% volume increase due to hydration) [7]. H_B_ showed water up-take values close to the highest ones reported for volumetric preparations, while the other two skinbooster preparations showed lower hydration ability, even with respect to the volumetric gels. It has to be considered that being injected at a superficial layer, a very high water uptake is undesirable as it may result in edema, which will reduce patient compliance [21,35]. However, the products that are proposed for more superficial injections need to be easily spreadable, this will ensure distribution of the gel in a larger area, and the water recall in the tissue will be more physiological. Finally, it is worth underlying that collected data do not directly translate into the fillers relative in vivo expansion at the injection site: compression forces exerted by the surrounding tissue, counteracting gel expansion, should also be considered for a more accurate prediction. The rheological data were also surprising since evidencing a huge difference in rigidity notwithstanding the specific proposed application as skinboosters: more than one order magnitude G’ variation was measured. Further, while J_V_ and H_B_ rigidity was, as expected, far lower than the one reported for the volumetric dermal fillers, the G’ value registered for R_V_ was higher than the majority of the volumetric gels and close to the R_Lift_ stiffness (R_Lift_ is the volumetric gel of the Restylane fillers family) [7]. A low rigidity is associated with high deformability under applied stress, thus reflecting in a reduced or even absent “palpability“ of the gels, which is crucial to obtain a natural-looking effect when gels are delivered in superficial skin layers [8,9,14,36,37].

Investigation of gel degradation profile is key since it is related to the in vivo longevity of the gels. All the samples showed sensitivity to BTH and ROS action thus ensuring in vivo resorbability. BTH was selected for this study because of the reported similarities to human hyaluronidases (HAses) [38,39] and because it is commercially available in pure form. BTH action is expected to have two effects: solubilization of the water-insoluble HA hydrogel and hydrolysis of the water-soluble HA chains (reduction of the average molecular weight). Despite the comparable rate of water-insoluble fraction solubilization, when equal volumes of the gels are injected, a longer in situ persistence can be predicted for H_B_, while J_V_ is expected as the shorter-lasting gel. This is related to the initial differences in total HA amount and insoluble hydrogel fraction that will resist enzymatic degradative conditions, based on the comparable degradation rate. Hydrodynamic analyses revealed that the molecular weight distribution of the soluble HA chains is widened during enzymatic hydrolysis. This is rationally related to the depolymerization of the water-soluble HA chains while progressive solubilization of the hydrogel may be responsible for the passage of longer polymeric chains to the solution. This would also explain the observed variation in the average molecular weight during incubation with the enzyme. The increase in c/M_n_ ratio (c is the water-soluble polymer concentration (mg/mL) and M_n_ is the number average molecular weight) proves, as expected, the increase in the polymer mol number in solution accompanying BTH action [40].

Data on the rheological behavior of the gels under in vitro oxidative stress conditions indicated markedly diverse sensitivity to ROS action. Specifically, based on collected data, the shorter permanence may be predicted for R_V_ while a more gradual loss of the clinical effect may be expected for H_B_ and, finally, J_V_ showed the highest resistance.

In vitro degradation studies either with ROS or with BTH serve as support to characterize the gels in comparable situations, however, the two phenomena are contemporary acting in vivo, besides the mechanical stress of face muscle movements, therefore the lifetime of the injected gels may be better derived through in vivo study. However, considering the more superficial delivery for skinboosters, and the intradermal penetration of UV radiation, sensitivity to ROS action, poorly investigated so far, has a key role for the gel stability when injected [41].

Skinboosters showed far lower resistance to ROS action than volumetric gels. Compared to the less sensitive volumetric gel, the most resistant skinbooster retained 50% rigidity in about the same time interval but in the presence of half the amount of ROS [7].

Cohesivity was recently defined as gel capacity to not dissociate [20]. Dermal fillers are lately investigated for their cohesive behavior referring to the pilot study by Sundaram and collaborators establishing a scale for gel rating [20]. Availability of cohesivity data on commercialized gels is helpful to clinicians in the selection of the most appropriate product to achieve the specific clinical objective. Fillers with higher cohesivity are considered a better choice for more superficial treatment. They allow spreadability and, therefore, a gel homogeneous distribution within the tissue, without fragmentation, thus avoiding palpability and even the eventually occurring formation of nodules [6,20,21]. H_B_ was the most cohesive. A better tissue integration pattern can be predicted for this gel based on its high cohesivity/low viscosity profile [6,20,21]. Results obtained in the framework of this study indicated that, despite the similar indication of use, the skinboosters here-characterized showed wide variability in terms of cohesivity (from 1 to 5) similar to our previous findings on volumetric preparations [7]. Even if the parameters affecting the gel’s cohesivity still need to be fully clarified, the strategy used for HA crosslinking may reasonably have an effect on gel’s behavior in this specific analysis.

Soft gels that are aiming at tissue revitalization should improve skin texture besides their effect in filling wrinkles. Their expected lower G’ and viscosity gives less performance than other gels to fill void volume, but this helps in reducing palpability and the Tyndall effect [19,21]. However, it can be argued that a biological effect is desirable beyond the physical effect. For this reason, we studied the gels in contact with human dermal fibroblasts. Data on the metabolic activity of cells, cultured on Tissue Cultured Polystyrene, in the presence of the hydrogels were demonstrated to be consistent with the CE approval of these class III medical devices, with an absence of any toxicity. Biological data showed a slight improvement in key biomarkers for H_B_ and R_V_ and a lower if none effect for J_V_. Specifically, elastin expression for H_B_ was superior with respect to the other treatments, and ColIV expression with respect to ColI was improved in R_V_ and H_B_ treatments. Comparison with data obtained for volumetric gels did not reveal a stronger biochemical effect for skinboosters on ColI and elastin production, however, the ratio collagen IV/Col I, besides elastin biosynthesis may support, as the final outcome, an improvement of flexible cutaneous basement membrane structure and, therefore, of skin (dermal tissue) quality and texture.

Biological data obtained on dermal fibroblasts at transcriptional and protein levels proved that lightly crosslinked HA-based gels, aiming at superficial treatments, were able to prompt collagen synthesis. Alteration in the extracellular matrix and especially collagen expression are generally associated with skin aging [42]. In this respect, improved biosynthesis of ECM proteins has a beneficial effect on skin/dermal rejuvenation procedures, besides wrinkles filling, leading to tissue hydration and consistency improvement [43]. It has been reported that integrins play a key function in cell adhesion as signaling receptors, acting as a bridge between ECM and cytoskeleton proteins in order to allow cell movement [44,45]. Activation of the integrin complex (α1β1) by an extracellular ligand (e.g., collagen, laminin and elastin) leads to specific intracellular signaling, involving phosphorylation and dephosphorylation reactions for matrix remodeling [46,47]. Our results allowed us to evaluate a beneficial effect of the analyzed samples on proteins matrix hypothesizing an involvement of the integrins receptor.

Finally, it is here demonstrated that biosynthetic pathways related to extracellular macromolecules are indeed affected by modified HA, even if this aspect was less investigated for these kinds of formulations generally used for their biomechanical effects.

Overall, it is worth underlying that the increasing number of in vitro studies characterizing commercial gels is surely useful to increase clinicians’ awareness in the selection and use of these gels and they are even more valuable in case of newly-developed gels for which clinical data are still lacking. However, attention has to be paid to translate these in vitro data to in vivo performance since additional aspects such as the mechanical properties of the tissue surrounding the implanted gel that can affect both hydration and projection capacity of the gels as well as individual inflammatory reactions and other in vivo mechanisms affecting degradation, and biological effect dependence on products in vivo degradation should be also considered.

## 4. Materials and Methods

### 4.1. Materials

Restylane Vital (R_v_) is *a* Q-Med AB product (Q-Med AB, Uppsala, Sweden) Juvederm Volite (J_V_) is distributed by *Allergan S.P.A*. (Pringy, France). Viscoderm Hydrobooster (H_B_) is distributed by *IBSA Farmaceutici* Italia srl (Lodi, Italy). They are HA-based dermal fillers intended for use as skinboosters. They all consist of BDDE-crosslinked HA, suspended in physiological medium. The total HA concentration, as reported in the package inserts, is 20 mg/mL for R_V__,_ 12 mg/mL for J_V_ and 25 mg/mL for H_B_. Bovine testicular hyaluronidase, BTH (EC 3.2.1.35), salt-free lyophilized powder with a specific activity of 890 U/mg was purchased from *Sigma-Aldrich S.R.L.* (Milan, Italy) (cat. N.H3884, lot. SLBF8562V). Dulbecco’s Phosphate Buffered Saline (PBS) without calcium and magnesium was purchased from *Lonza Sales Ltd.*, Switzerland (cat. N.BE17-516F, lot. N.3MB191). Hydrogen peroxide, 30% *w*/*w* in water was purchased from Sigma Aldrich *S.R.L.* (Milan, Italy), cat. N.H1009. Copper (II) sulfate (≥99%) Fluka, cat. N.61230, was purchased from Sigma Aldrich *S.R.L.* (Milan, Italy).

### 4.2. Biophysical and Chemical Characterization

#### 4.2.1. Soluble Fraction Quantification and Hydrodynamic Characterization

Filler soluble fraction was quantified as reported elsewhere [7,8]. Briefly, each filler was diluted to 4 mg/mL in PBS (1.0 mL final volume). The resulting suspension was kept under stirring (1000 rpm) for 24 h at 37 °C. The sample was then centrifuged at 13,000× *g* for 5 min, and the supernatant was removed and filtered on 0.22 μm. Filtered samples were then quantitatively analysed for the HA content by carbazole. Based on the total HA concentration indicated in the package inserts, the amount of water-insoluble HA (mg/mL) in each gel was calculated. Hydrodynamic characterization of gel’s soluble fractions was also performed by using the SizeExclusion Chromatography–Triple Detector Array (SEC-TDA) equipment by Viscotek (Malvern Panalytical). Specifically, several aliquots of filtered samples, obtained as described above, were diluted at concentrations suitable for the chromatographic analysis.

The hydrodynamic parameters for the soluble fraction of the gels were determined by the Size Exclusion Chromatography-Triple Detector Array (SEC-TDA) equipment by Viscotek (Viscotek, Malvern, UK). A detailed description of the SEC-TDA system, of its potential to analyse biopolymers such as HA, and of the analysis conditions are reported elsewhere [40]. Sample’s molecular weight (*M*_w_, M_n_, *M*_w_/M_n_), molecular size (hydrodynamic radius—R_h_) and intrinsic viscosity ([η]) distributions were derived. The Mark–Houwink–Sakurada (MHS) curve (log[η] vs. log*M*_w_) was directly derived for each sample.

#### 4.2.2. ^1^H-NMR Analyses

Gels were investigated for the BDDE/HA content by ^1^H-NMR, as previously described, with slight modifications [25]. Briefly, gels were diluted to 4 mg/mL in HCl 0.01 M and incubated for 72 h at 70 °C under stirring (400 rpm). The samples were neutralized by adding Na_2_HPO_4_ and lyophilized. The dried samples were dissolved in D_2_O and ^1^H-NMR analyses were performed using a Bruker DRX-400 (^1^H NMR: 400 MHz) instrument at 298 K. Data were processed using the data analysis packages integrated with Bruker TopSpin^®^ 4.0.5 software.

#### 4.2.3. Hydration Capacity

Gels were diluted to 4 mg/mL in PBS and incubated overnight in thermoshaker at 37 °C under stirring for 16 h. After centrifugation (13,000× *g*, 5 min) and supernatant removal, the pellet was weighed obtaining the hydrated sample mass (g), corresponding to the hydrated sample volume (mL) (density equal to 1 g/mL). The hydration extent of each gel was calculated as:

(1) $$\mathrm{gel}’s\;\mathrm{hydration}\;\mathrm{capacity}\left(\frac{\mathrm{mL}}{\mathrm{mL}}\right)=\frac{\mathrm{hydrated}\;\mathrm{sample}\;\mathrm{volume}\;\;(\mathrm{mL})}{\mathrm{initial}\;\mathrm{sample}\;\mathrm{volume}\;\left(\mathrm{mL}\right)}$$

Such values represent the volume expansion for each formulation when allowed to reach the equilibrium swelling in PBS.

#### 4.2.4. Rheological Characterization

Rheological measurements were performed as reported elsewhere [7,8] with slight modifications. A Physica MCR301 oscillatory rheometer (Anton Paar, Ostfildern, Germany) equipped with a parallel plate geometry, 25 mm plate diameter (R_v_, J_v_) and 50 mm (H_B_), and Peltier temperature control was used. Measurements were performed at 37 °C. Oscillation frequency sweep tests were carried out over a frequency range from 0.159 to 10 Hz (a range of frequencies considered physiologically relevant for the specific application), at a constant strain selected within the linear viscoelastic range (0.1%). G′ and G″ were measured and reported as a function of frequency. Complex viscosity values were also registered in the frequency range exploited. Representative curves are reported.

#### 4.2.5. Stability to Degradation

Gels were evaluated for their sensitivity to degradation due to ROS- and BTH-action.

Stability of the gels to ROS action was studied using the H_2_O_2_/Cu^2+^ system for generating radicals. Experiments were carried out as previously reported, with minor modification [7]. Briefly, aqueous solutions of H_2_O_2_ and CuSO_4_ were added to each gel to have H_2_O_2_ 187 mM and CuSO_4_ 1.87 mM while diluting the gel 1:1.3 (*w*/*w*). The suspensions were mixed and rapidly placed on the lower plate of the rheometer. A PP25 geometry, (25 mm plate diameter) was used. A time oscillatory test was carried out at 37 °C. Specifically, the storage modulus value of the samples was measured as a function of the time while maintaining constant the frequency (1.59 Hz) and the strain (0.1%). The delay between the addition of the H_2_O_2_/Cu^2+^ system to the samples and the acquisition of the first G’ value was 1–3 min. For each gel, the same experiment was performed adding water in place of the H_2_O_2_/Cu^2+^ system (control). Degradation was monitored by measuring the G’ decrease (% in respect to the control) as a function of the incubation time (up to 8′) with the ROS generating system. Experiments were carried out in duplicate.

Sensitivity of the gels to enzymatic degradation was evaluated as previously reported with slight modification [7,8,25]. Specifically, the amount (mg/mL) of HA solubilized due to BTH action was determined as a measure of degradation. Gels were diluted in PBS to 4 mg/mL final concentration and incubated in the presence of BTH 2 U/mL (3 h) and 5 U/mL (3 h and 6 h) at 37 °C under stirring. Testing was interrupted by boiling the sample for 10 min to inactivate the enzyme. Samples were then centrifuged at 13,000× *g* for 5 min. The supernatant was removed, filtered on 0.22 μm, opportunely diluted in water and then quantitatively analyzed for the HA content by carbazole assay. The amount of soluble HA already existing in the gel (determined as indicated in Section 4.2.1) was subtracted to obtain the amount of HA solubilized due to BTH action. Further, the water-soluble fraction derived from the less and the most drastic enzymatic hydrolysis conditions tested underwent hydrodynamic characterization using the SEC-TDA system (see also Section 4.2.1).

#### 4.2.6. Cohesivity

Product cohesivity was evaluated following the validated protocol reported by Sundaram and collaborators [20]. Specifically, 1 mL of gel was stained with 10 µL of toluidine blue (0.1%) and filled in a 1 mL syringe. The sample was extruded in a 1 L beaker with 700 mL of water mQ, while stirring (160 rpm) with the aid of the magnetic stirred (2.5 cm). Photos at diverse time intervals and videos were taken. Cohesivity was evaluated independently by 4 raters that assigned, for each sample, at each time, a value of cohesivity (from 1 to 5) referring to the Gavard–Sundaram Cohesivity Scale [20]. Results were reported as the mean score ± SD.

### 4.3. Biological Evaluation

#### 4.3.1. Cell Cultures

A human dermal fibroblast cell line immortalized with hTERT (HDF cells, BJ-5ta, ATCC CRL-4001) was cultured in a 4:1 mixture of Dulbecco’s Modified Eagle Medium (DMEM) and Medium199 supplemented with 0.01 mg/mL hygromycin B and 10% (*v*/*v*) FBS. All materials for HDF culture were purchased from Gibco and Invitrogen. The cells were grown on tissue culture plates using an incubator with a humidified atmosphere (95% air/5% CO_2_ *v*/*v*) at 37 °C.

#### 4.3.2. Cell Viability (MTT Test)

MTT test was accomplished according to La Gatta et al., 2018. Briefly, cells were seeded at a density equal to 2∙ × 10^4^ cells/cm^2^ in 12 wells. Twenty-four hours after seeding, the treatments were added to the medium at 0.16% *w*/*w* concentration. After 24 h from gels addition, the cells were observed at inverted optical microscope (MO). Then, cell viability was assessed by measuring the reduction of the tetrazolium dye 3-(4,5-dimethylthiazol-2-yl)-5-(3 carboxymethoxyphenyl)-2-(4-sulfophenyl)-2H–tetrazolium (MTT). Medium was removed and cells were treated three times with PBS to remove any residual suspended hydrogels before adding the MTT solution. Cell viability in presence of fillers was reported with respect to untreated cells (viability %).

#### 4.3.3. Type I Collagen (COLIA1), Type IV Collagen (COLIVA1), Elastin (ELS) and Integrins (INTα1 and β1) mRNA Quantification Using qRT-PCR Analyses

Total RNA was extracted using TRIzol RNA Isolation Reagents (Thermofischer scientific, Waltham, MA USA), and reverse transcribed by Reverse Transcription System Kit (Promega, Milan, Italy). Quantitative real-time polymerase chain reactions (qRT-PCR) were performed in duplicate for all genes of interest using IQ ™ SYBR^®^ Green Supermix (Bio-Rad Laboratories, Milan, Italy) and internal control (glyceraldehyde-3-phosphate dehydrogenase, (GAPDH) housekeeping gene). Results are expressed as fold change (2^−ΔΔCt^ method) in treated cells vs. untreated cells (the control), and normalized to transcript levels of housekeeping gene [48]. qRT-PCR was performed using custom primers reported in Table 3.

#### 4.3.4. Western Blotting for Collagen Type 1, Elastin, and Actin

Western blotting analyses were performed after 48 h of treatment. Cells were lysed in Radio-Immunoprecipitation Assay (RIPA buffer 1×; Cell Signaling Technology) and intra-cellular protein concentration was determined through the Bradford method. Specifically, 30 μg of proteins for each sample were resolved on a 10% SDS–PAGE gel and transferred onto nitrocellulose membrane (GE, Amersham, UK). Then, the membrane was blocked by 5% non-fat milk in Tris-buffered saline and 0.05% Tween-20 (TTBS) for 30 min and primary antibodies against Elastin (Santa Cruz, Dallas, TX, USA), Col I (Elabscience, Houston, TX, USA) and Col IV (Abcam, Cambridge, UK) were diluted 1:500 and incubated overnight at 4 °C. Secondary antibodies horseradish peroxidase-conjugated donkey anti-mouse and goat anti-rabbit were diluted 1:5000 and incubated for 2 h at room temperature. Anti-β-Actin antibody used at 1:1000 dilution was used as the loading control. The signal was detected using the ECL system (Chemicon-Millipore, Milano, Italy) and the semi-quantitative analyses of protein expression were carried out with the ImageJ program.

### 4.4. Statistical Analysis

Unless otherwise indicated, each experiment was performed at least in triplicate and results are reported as the mean value ± standard deviation. Data were statistically evaluated by performing One-way ANOVA tests followed by post hoc correction for multiple comparison. The level of significance was fixed at 0.05.

## 5. Conclusions

Biophysical and biochemical parameters were derived for three crosslinked HA-based gels proposed for intradermal injection as skinboosters. Collected data indicated H_B_ as the gel with the highest concentration in insoluble HA and the longer soluble HA chains and with the highest water uptake, suggesting deeper hydration properties. H_B_ showed the lowest rigidity/viscosity and the highest cohesivity. J_V_ was found to be the most resistant to degradation by ROS while longer permanence in the presence of BTH was recorded for H_B_. HA-based samples determined a significant improvement of matrix biomarkers expression at the transcriptional level and strong upregulation of integrins was found for J_V_ and H_B_ treated fibroblasts. However, Western blotting analyses showed only a slight boosting effect of gels on Human Dermal Fibroblasts in terms of Col IV/Col I and elastin production.

Comparison with conventional volumetric formulations revealed differences far lower than expected in relation to composition, hydration capacity and biochemical effects. However, except for R_V_, rigidity was decisively lower indicating higher deformability as the most important requirement for more superficial implantation.

These results are thought to be useful to practitioners for improving the use of these gels and valuable to adjust gel design towards even more specific performance.

## Figures and Tables

**Table 1:** Amount of water-soluble and water-insoluble.

HA (mg/mL)	R_V_	J_V_	H_B_
**total**	20	12	25
**Water-soluble**	4.7 ± 0.6	4.0 ± 0.6	4.2 ± 0.9
**Water-insoluble**	15.4 ± 0.6	8.0 ± 0.6	20.8 ± 0.9
**Soluble HA (wt%)**	23 ± 3	33 ± 5	17 ± 4

HA in each gel and hydrodynamic parameters for the water-soluble HA fractions as derived from the Size Exclusion Chromatography-Triple Detector-Array (SEC-TDA) analyses. The total HA concentration reported is the value indicated in the product’s package insert.

**Table 2:** Weight average molecular weight (M_w_), numeric average molecular weight (M_n_), polydispersity index (M_w_/M_n_), intrinsic viscosity ([η]) and hydrodynamic radius (R_h_).

Sample	Hydrodynamc Parameters for the Soluble HA Fractions				
	M_W_ (kDa)	M_n_ (kDa)	M_W_/M_n_	[η] (dL/g)	R_h_ (nm)
**R_V_**	266 ± 29	109 ± 19	2.5 ± 0.6	5.7 ± 0.1	26.8 ± 1.1
**J_V_**	161 ± 21	49 ± 5	3.3 ± 0.2	3.4 ± 0.2	17.9 ± 1.0
**H_B_**	525 ± 23	305 ± 55	1.7 ± 0.2	7.5 ± 0.4	37.2 ± 1.7

**Table 3:** Primers sequence used for qRT-PCR analyses.

Gene Name (Symbol)	PCR Primer Sequence 5′-3′	Amplicon Length (bp)
Glyceraldehyde3-phosphate dehydrogenase (GAPDH)	TTCCACGGCACAGTCAA GCAGGTCAGGTCCACAA	115
Type I collagen (COLIA1)	CCAGAAGAACTGGTACATCA CCGCCATACTCGAACTGGAA	103
Type IV collagen (COLIVA1)	GGATCGGCTACTCTTTTGTGATG AAGCGTTTGCGTAGTAATTGCA	104
Elastin (ELS)	AGGTGTATACCCAGGTGGCGTGCT CAACCCCTGTCCCTGTTGGGTAAC	105
α-1 integrin (INTα1)	TCGCCAGCTTTGGAAGTCAT ATGTACTGGAGTTGGGCAGC	108
β-1integrin (INTβ1)	ACTGTGATGCCGTATATTAGCAC GATATGCGTTGCTGACCAACA	110

## Supplementary Materials

The following are available online at https://www.mdpi.com/article/10.3390/ijms22116005/s1.
