# Supplementary material for: Hyaluronan Hydrogels for Injection in Superficial Dermal Layers: An In Vitro Characterization to Compare Performance and Unravel the Scientific Basis of Their Indication
Source: Int J Mol Sci. 2021 Jun 2;22(11):6005. doi: 10.3390/ijms22116005 (PMC8199639; doi:10.3390/ijms22116005)
Supplement: Supplementary file 1 [file ijms-22-06005-s001.zip › ijms-1157884-supplementary.pdf]

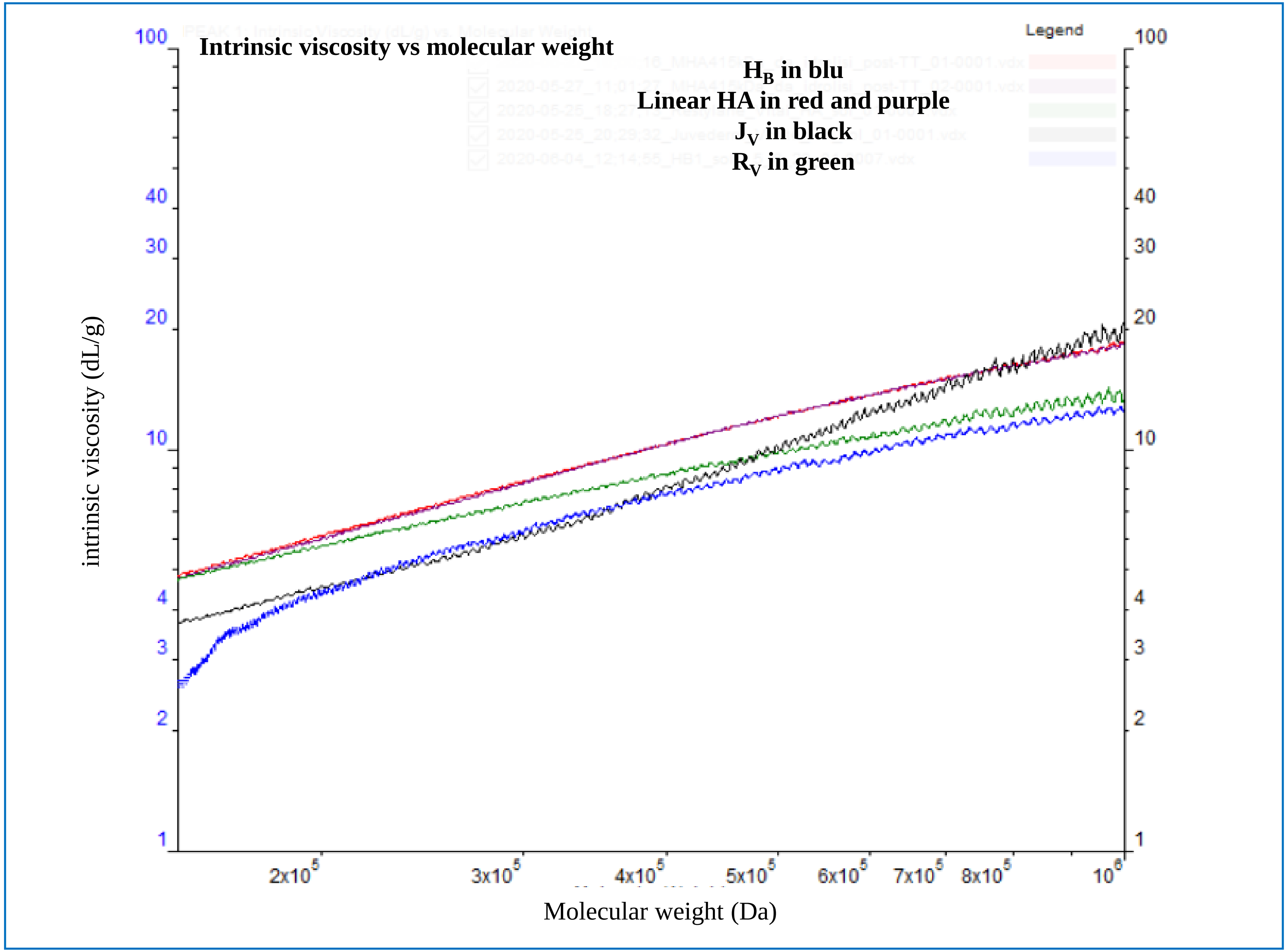

Figure S1. Superimposition of the MHS curves (intrinsic viscosity *vs* molecular weight) for the soluble fractions of the investigated samples and for a linear HA sample.
